# Very early withdrawal from treatment in patients starting peritoneal dialysis

**DOI:** 10.1080/0886022X.2017.1419965

**Published:** 2018-01-03

**Authors:** Qimei Luo, Xi Xia, Zhenchuan Lin, Jianxiong Lin, Xiao Yang, Fengxian Huang, Xueqing Yu

**Affiliations:** a Department of Nephrology, The First Affiliated Hospital, Sun Yat-sen University, Guangzhou, China;; b Key Laboratory of Nephrology, Ministry of Health, Guangzhou, China

**Keywords:** Very early withdrawal, peritoneal dialysis, end-stage renal disease, risk factors, chronic kidney disease

## Abstract

**Introduction:** Very early withdrawal from treatment in patients undergoing peritoneal dialysis (PD) is an increasingly important, but poorly understood, issue. Here, we identified the reasons and risk factors for very early withdrawal from PD.

**Methods:** Incident PD patients from The First Affiliated Hospital of Sun Yat-sen University above 18 years who started treatment between January 1 2006 and December 31 2011 were included. Cessation of PD therapy within the first 90 days after beginning dialysis was classified as very early withdrawal.

**Results:** Totally 1444 patients were enrolled. Of these, 71 (4.9%) withdrew from PD therapy during the first 90 days. Primary reasons for very early withdrawal included death (34 patients, 47.9%), transplantation (21 patients, 29.6%) and transfer to hemodialysis (14 patients, 19.7%). The leading reasons for death were cardiovascular and infectious disease, accounting for 41.2% (14 patients) and 23.5% (8 patients) of total deaths, respectively. Dialysate leakage (six patients, 42.9%) and catheter dysfunction (five patients, 35.7%) were the main reasons for transfer to hemodialysis. In multivariate analysis, predictors for very early PD withdrawal were older age (per decade increasing; hazard ratio [HR], 1.22; 95% confidence interval [CI], 1.03–1.45; *p* = .019), higher systolic blood pressure (per 10 mmHg increasing; HR, 1.35; 95% CI, 1.20–1.50; *p* < .001), lower hemoglobin (per 10 g/l increasing; HR, 0.67; 95% CI, 0.57–0.78; *p* < .001), lower high-density lipoprotein cholesterol (HR, 0.24; 95% CI, 0.10–0.54; *p* = .001) and lower residual urine volume (per 100 ml/d increasing; HR, 0.90; 95% CI, 0.84–0.95; *p* = .001).

**Conclusions:** Death was the primary reason for very early withdrawal from PD. Risk factors for very early withdrawal from PD were older in age, had higher systolic blood pressure, lower hemoglobin, lower high-density lipoprotein cholesterol and lower residual urine volume.

## Introduction

The number of patients with end-stage renal disease (ESRD) is significantly increasing worldwide. The projected number of people receiving renal replacement therapy (RRT) will reach 5.439 million by 2030, with the most growth in Asia [1]. Peritoneal dialysis (PD) has emerged as an important modality of RRT due to its simplicity and minimal requirements for technical support and electricity [2]. Although there have been numerous technical advances in dialysis therapy, many patients still withdraw from PD treatment. Rates of withdrawal from PD therapy ranged from 19.8 to 54.8% depending on different population and study period [3–6]. Additionally, some patients still withdraw from PD treatment early. Descoeudres et al. reported that 54.8% of patients withdrew from PD therapy and of these, 29% stopped treatment within the first six months after PD catheter insertion [3]. Another retrospective study from Japan found that 6.5% of patients discontinued PD treatment within the first three months after initiation of dialysis [7]. It’s important to identify patients who very early withdraw from treatment in order to inform and arrange dialysis program efficiently, improve the procedure of peritoneal catheter implantation and allocate resources rationally. Factors associated with the risk of very early withdrawal from PD treatment may help clinicians to identify patients.

There are few studies focused on very early dropout from PD treatment. Several studies have explored risk factors associated with withdrawal from PD during the whole treatment, and reported risk factors include increased patient age, hernia formation during PD therapy and patients treated by hemodialysis (HD) before PD [3,8,9]. Importantly, using a time-dependent approach to analyze treatment failure in PD patients, Kolesnyk et al. found that during different periods of treatment, the reasons for cessation of PD and the risk factors for dropout were different [7]. Thus we conducted a retrospective cohort study to identify the reasons and risk factors for very early withdrawal from treatment in patients starting PD.

## Methods

### Participants

All incident patients >18 years of age, who received PD therapy from January 1 2006 to December 31 2011 were recruited from a single PD center of the First Affiliated Hospital of Sun Yat-sen University. Patients who refused to give written consent were excluded from this study.

### Study protocol

This was a retrospective study conducted in a single PD center. Baseline demographic, clinical and biochemical data were collected at the initiation of PD therapy. The primary end point was withdrawal from PD, which was defined as the composite end point of a permanent switch to HD, transplantation, died due to any cause or gave up renal replacement treatment. Patients were classified as having undergone very early PD withdrawal if the primary end point was reached within 90 days after initiation of PD therapy. Hypertension was defined as taking antihypertensive drugs or having two separate blood pressure measurements ≥140/90 mmHg. Diabetes mellitus was noted in patients who received insulin or oral hypoglycemic agents and/or who were diagnosed with type 1 or type 2 diabetes mellitus. Cardiovascular disease (CVD) was defined as having a history of angina pectoris, myocardial infarction, angioplasty, coronary artery bypass, heart failure or stroke [10]. Biochemical data, including levels of hemoglobin, serum albumin, serum uric acid, serum creatinine, albumin-corrected calcium, serum phosphorus, alkaline phosphatase, triglycerides, total cholesterol, high-density lipoprotein cholesterol (HDL-C) and low-density lipoprotein cholesterol (LDL-C) were measured in the First Affiliated Hospital of Sun Yat-sen University. Cardiovascular mortality was defined as death due to acute myocardial infarction, atherosclerotic heart disease, cardiac arrhythmia, cardiomyopathy, congestive heart failure, cardiac arrest, cerebrovascular accident (including intracranial hemorrhage), ischemic brain damage, anoxia encephalopathy and peripheral vascular disease [10]. All patients were followed up for at least three months. The study protocol was approved by the Human Ethics Committee of The First Affiliated Hospital, Sun Yat-sen University. All patients provided written informed consent before study entry.

### Statistical analysis

Participants were divided into two groups according to whether or not they underwent very early withdrawal from PD therapy. We then compared the demographic, clinical and biochemical data from patients between two groups. Results are described as frequencies and percentages for categorical data, means and standard deviations (SDs) for approximately normally distributed continuous variables and interquartile ranges for skewed continuous variables. Chi-squared or Mann-Whitney tests were used to measure significance differences in categorical or continuous factors between two groups. Patients were considered censored if they recovered renal function, lost to follow up within the first 90 days of PD therapy or reached the end of the follow up period. Cox regression models were used to identify the risk factors for very early withdrawal from PD therapy. The multivariate Cox regression model was performed using eligible variables that demonstrated significant association with very early withdrawal from PD therapy from the univariate analysis or were considered to reflect important clinical concerns. The results were expressed as the hazard ratio (HR) and 95% confidence interval (CI). Statistical analyses were conducted using SPSS, version 21.0 (SPSS Inc, Chicago, IL) and in all cases, a significance of *p* < .05 was considered statistically significant.

## Results

### Patient characteristics

**Figure 1.**
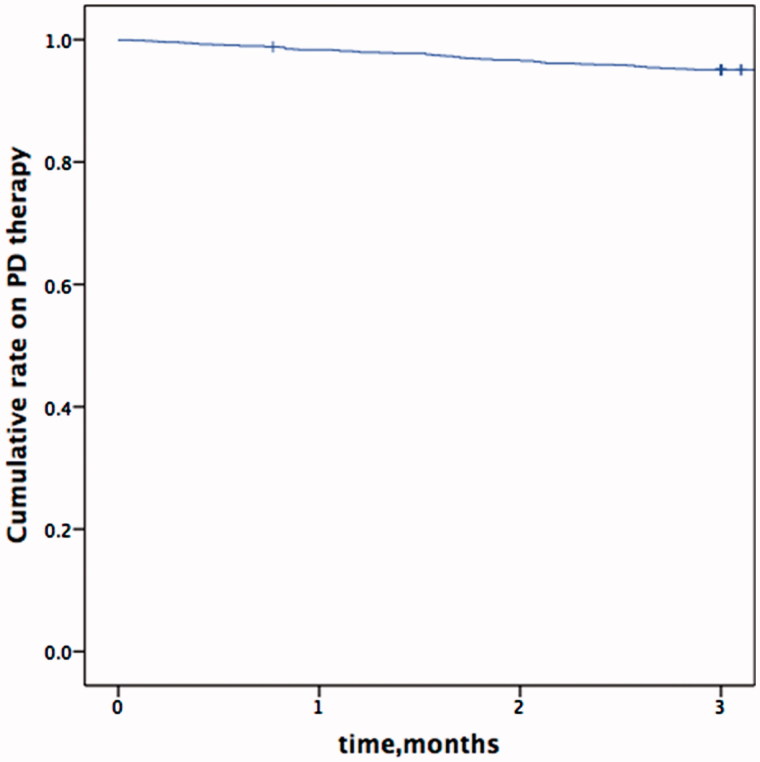
Kaplan-Meier curve depicting rate on peritoneal dialysis during the first 90 days of therapy.

Of the 1456 patients who initiated PD therapy during the study period, 12 were excluded due to refusal to provide written consent. A final sample group of 1444 patients was therefore analyzed. The mean age at the start of PD therapy was 48.6 ± 15.7 years; 57.8% of the subjects were men, 25.6% had diabetic mellitus and 26.8% had a history of CVD. A total of 56 patients received maintenance HD before the start of PD therapy and 10 accepted kidney transplantation before PD therapy initiation. Of the enrollment subjects, 71 (4.9%) withdrew from PD therapy within the first 90 days of dialysis. The Kaplan-Meier curve depicting the rate on PD during the first 90 days of therapy is shown in Figure 1. A comparison of data on demography and baseline clinical variables between the patients very early withdrew from PD and those that remained on PD are listed in Table 1.

**Table 1.** Patients characteristics according to occurrence of very early withdrawal from PD therapy.

Covariate^b^	Very early withdrawal (*n* = 71)	No withdrawal (*n* = 1373)	*p* value
Age at PD initiation (year)^a^	55 ± 20.7	47 ± 15.4	.048
Man, *n* (%)	36 (50.7)	793 (57.8)	.241
Comorbidity conditions, *n* (%)			
Diabetes mellitus	19 (26.8)	352 (25.6)	.833
Cardiovascular disease	16 (22.5)	379 (27.6)	.350
Hypertension	52 (73.2)	876 (63.8)	.106
Malignancy	4 (5.6)	22 (1.5)	.042
Systolic BP (mmHg)	151 ± 22	137 ± 19	<.001
Diastolic BP (mmHg)	82 ± 16	84 ± 14	.631
Therapy before PD initiation, *n* (%)			
Maintenance hemodialysis	2 (2.8)	54 (3.9)	.635
Transplantation	1 (1.4)	9 (0.7)	.397
Laboratory variables			
Hemoglobin (g/L)^a^	77.5 ± 19.2	95.3 ± 18.5	<.001
Serum albumin (g/L)^a^	33.7 ± 5.3	36.2 ± 4.5	<.001
Serum uric acid (mg/dL)^a^	7.19 ± 2.31	7.15 ± 1.52	.622
Serum creatinine (mg/dL)^a^	9.06 ± 3.61	9.84 ± 3.41	.024
Serum calcium (mg/dL)^a^	8.39 ± 0.79	8.87 ± 0.83	<.001
Albumin correct calcium (mg/lL)^a^	8.90 ± 0.80	9.17 ± 0.78	<.001
Serum phosphorus (mg/dL)^a^	5.56 ± 2.03	5.18 ± 1.39	.316
i-PTH (pg/ml)^a^	340.6 ± 262.5	337.8 ± 301.5	.667
Alkaline phosphatase (U/L)^a^	81.8 ± 50.6	73.6 ± 39.9	.371
Triglycerides (mmol/L)^a^	1.79 ± 0.93	1.69 ± 1.12	.108
Total cholesterol (mmol/L)^a^	4.69 ± 1.24	4.95 ± 1.18	.071
HDL-C (mmol/L)^a^	0.94 ± 0.35	1.17 ± 0.35	<.001
LDL-C (mmol/L)^a^	2.74 ± 0.95	2.86 ± 0.85	.258
24 h urine output (ml/d)^a^	480 ± 351	766 ± 540	<.001

a Data are presented as mean ± standard deviation.

b Conversion factors for units: serum uric acid in mg/dL to umol/L, ×59.48; serum creatinine in mg/dL to umol/L, ×88.4; serum calcium in mg/dL to mmol/L, × 0.2495; serum phosphorus in mg/dL to mmol/L, × 0.3229; triglycerides in mmol/L to mg/dL, × 88.496; total cholesterol, HDL-C and LDL-C in mmol/L to mg/dL, × 38.669.

PD: peritoneal dialysis; BP: blood pressure; i-PTH: immunoreactive parathyroid hormone; HDL-C: high-density lipoprotein cholesterol; LDL-C: low-density lipoprotein cholesterol.

### Reasons for very early withdrawal from PD therapy

From our patient cohort, 71 subjects withdrew from PD therapy within the first 90 days after beginning treatment; 34 (49.7%) died, 21 (29.6%) received kidney transplant and 14 (19.7%) were transferred to HD (Table 2). The remaining two patients declined additional renal replacement therapy. Death due to cardiovascular disease and infectious diseases accounted for 41.2 and 23.5% of deaths, respectively. In addition, four patients died because they declined to receive further life support. Dialysate leakage and catheter dysfunction were the main reasons for transfer to HD (Table 3).

**Table 2.** Reasons for withdrawal from PD during the first 90 days of dialysis.

	Number (percentage)
Death	34 (47.9)
Cardiovascular diseases	14 (41.2)
Acute myocardial infraction	5 (35.7)
Sudden cardiac death	5 (35.7)
Congestive heart failure	2 (14.3)
Cerebrovascular disorder	2 (14.3)
Infectious diseases	8 (23.5)
Pulmonary infection	5 (62.5)
Intracranial infection	1 (12.5)
Intestinal infection	1 (12.5)
Peritoneal dialysis-associated peritonitis	1 (12.5)
Malignancy (lymphoma)	1 (2.9)
Declined further life support	4 (11.8)
Gastrointestinal bleeding	3 (8.8)
Pulmonary embolism	1 (2.9)
Suicide	1 (2.9)
Unknown cause	2 (6.0)
Transplantation	21 (29.6)
Transfer to HD	14 (19.7)
Gave up renal replacement treatment	2 (2.8)

PD: peritoneal dialysis; HD: hemodialysis.

**Table 3.** Reasons for transfer to HD in patients starting PD.

	Number (percentage)
Dialysate leakage	6 (42.9)
Pleura-abdominal fistula	5 (83.3)
Scrotal hydrocele	1 (17.7)
Catheter dysfunction	5 (35.7)
Catheter displacement	3 (60.0)
Diminished outflow volume	2 (40.0)
Patients willing	2 (14.3)
Lactic acidosis	1 (7.2)

HD: hemodialysis; PD: peritoneal dialysis.

### Risk factors for very early withdrawal from PD therapy

Univariable analysis revealed that increased age, higher systolic blood pressure (SBP) and higher levels of serum phosphorus were significantly associated with very early withdrawal from PD therapy (*p* < .05). Conversely, lower levels of hemoglobin, serum albumin, serum calcium, serum HDL-C and the volume of 24 h urine output were associated with increased risk of very early withdrawal from PD therapy (*p* < .05). Based on a multivariable Cox regression analysis (Table 4), we found that significantly influential variables for very early dropout form PD were older age, higher SBP, lower levels of hemoglobin, lower levels of serum HDL-C and lower volume of 24 h urine output. When patients who withdrew from PD due to transplantation (*n* = 21) were excluded from the analysis, older age, higher SBP, lower hemoglobin and lower 24 h urine output were still found to be risk factors for very early withdrawal from PD therapy (Supplementary Table 1).

**Table 4.** Predictors for PD withdrawal during the first 90 days of dialysis.

	Univariate analysis		Multivariate analysis	
Variable	HR (95% CI)	*p* value	HR (95% CI)	*p* value
Age (per decade increasing)	1.22 (1.05,1.42)	.009	1.22 (1.03,1.45)	.019
Male sex	1.32 (0.83,2.11)	.239	1.25 (0.76,2.04)	.381
Diabetes mellitus (yes/no)	1.06 (0.63,1.80)	.827	0.74 (0.42,1.31)	.301
Cardiovascular disease (yes/no)	0.77 (0.44,1.34)	.356	0.58 (0.32,1.06)	.076
Systolic pressure (per 10mmHg increasing)	1.38 (1.24,1.53)	<.001	1.35 (1.20,1.50)	<.001
Hemoglobin (per 10g/L increasing)	0.61 (0.54,0.69)	<.001	0.67 (0.57,0.78)	<.001
ALB (per 1g/L increasing)	0.90 (0.86,0.94)	<.001	0.99 (0.93,1.05)	.689
Serum calcium (per 1 mg/dL increasing)	0.11 (0.04,0.27)	<.001	0.83 (0.25,2.78)	.758
Serum phosphorus (per 1mg/dL increasing)	1.64 (1.05,2.57)	.031	0.96 (0.60,1.55)	.878
HDL-C (per 1 mmol/L icreasing)	0.10 (0.04,0.23)	<.001	0.24 (0.10,0.54)	.001
24 h urine output (per 100ml/d increasing)	0.88 (0.83,0.93)	<.001	0.90 (0.84,0.95)	.001

PD: peritoneal dialysis; HR: hazard ratio; CI: confidence interval; ALB: albumin; HDL-C: high-density lipoprotein cholesterol.

## Discussion

In the present study, we analyzed the reasons and risk factors for withdrawal from PD therapy in the first 90 days after beginning treatment. We found that in our study cohort, 4.9% of incident PD patients discontinued PD treatment during the first 90-day period. Death was the leading cause of very early withdrawal from PD therapy, with most mortalities resulting from cardiovascular and infectious diseases. In contrast to our study, a nationwide survey in Japan reported that transfer to HD was the main reason for withdrawal from PD therapy and of the patients who died during PD, cerebrovascular accident was the leading cause of mortality [11]. We postulate that these results differ from ours partly due to differences in the time of withdrawal. Specifically, our study focused on patients who withdrew from PD therapy during the first 90 days after initiation of treatment, whereas Kawaguchi et al. [11] did not restricted the time of withdrawal. Further, we postulate that over the course of prolonged PD, more patients are likely to transfer to HD due to ultrafiltration failure or to avoid the development of encapsulating peritoneal sclerosis [12,13]. Notably, when restricted to the first 90 days of treatment, similar to our findings, the main reason for PD discontinuation during the first 90 days of treatment in the Kolesynk et al. [7] was death.

Here, we observed that dialysate leakage and catheter dysfunction were the main reasons for transfer to HD. A Swiss study [3], which analyzed the importance of early treatment failure in PD within the first six months, found that catheter and psychosocial problems were most commonly responsible for early treatment failure. Another retrospective study using data from the French Language Peritoneal Dialysis Registry (RDPLF) similarly showed that catheter dysfunction and psychosocial reasons were the main reasons for switch to HD within the first six months of PD [8]. PD-related peritonitis, ultrafiltration failure and encapsulating peritoneal sclerosis were reported to be the primary reasons for late transfer to HD [14]. Catheter problems contributed more often to early switch to HD than to late switch to HD. Early dialysate leakage was likely due to the poor condition of the abdominal wall and diaphragm, as either diaphragmatic defects or lymphatic transport across the diaphragm results in peritoneopleural leakage. It is important to enhance evaluation of the condition of the abdominal wall and the diaphragm and perfect the procedure of operation in order to avoid early transfer to HD.

The results of the present study suggest that within the first 90 days after beginning PD treatment, very early withdrawal is likely related to worsened patient’s condition at the time of therapy initiation. Patients who withdrew very early in our study were older in age, displayed markers of poor nutritional status (lower albumin levels and lower serum creatinine levels) and displayed several symptoms of severe uremic syndrome (e.g., anemia, hypocalcemia and decreased volume of 24 h urine output). These characteristics likely contribute to very early withdrawal from PD therapy, particularly for those who died during this time period.

Several studies examining dialysis withdrawal have shown an increased risk in older age patients [15–18]. An analysis of the Australia and New Zealand Dialysis and Transplant (ANZDATA) report found that older age was one of the predictors for dialysis withdrawal in the first year of initiation of dialysis [16]. Another cohort study from the United States showed that during the first year of dialysis, the rates of mortality and transferred to HD increased with age [17]. Our study similarly revealed that older age remains an important risk factor for very early withdrawal from PD.

Few studies have focused on the relationship between SBP and dialysis withdrawal, although a number of investigations have characterized how blood pressure affects mortality in dialysis patients. Of note, Udayaraj et al. reported a variable relationship between blood pressure and mortality in PD and showed that greater SBP was associated with decreased mortality in the first year, whereas it was associated with increased long-term mortality [19]. Another observational study founded that an SBP <111 mmHg in PD patients was associated with a higher mortality risk and conversely a protective effect was observed with a SBP >120 mmHg [20]. Here, we found that higher SBP was a risk factor for very early PD cessation. The definition of withdrawal in the present study included death, transfer to HD and any other cessation of PD therapy. In contrast, the studies mentioned above focused only on mortality and the time of withdrawal was not limited. Thus impact of SBP on dialysis withdrawal remains unclear and further studies should be conducted to investigate the exact relationship between SBP and dialysis withdrawal.

A recent study found that low hemoglobin levels were associated with mortality, as well as PD to HD switch in PD patients [17]. Additionally, low hemoglobin levels correlated with withdrawal from PD therapy during a four year study [21]. Similarly, we found that even during the very early period, low hemoglobin levels increased the risk for PD cessation. Another risk factor for very early withdrawal from PD therapy in our study was low HDL-C levels. Notably, low HDL-C levels have been associated with incident cardiovascular events [22] and for individuals without previous cardiovascular conditions, those with lower HDL-C levels had a higher risk of cardiovascular mortality, as compared to subjects with normal HDL-C levels [23]. Low HDL-C in chronic kidney disease patients was associated with an increase in intermediate monocytes, which was a type of monocyte with poor cholesterol efflux capacity [24]. Cholesterol efflux from peripheral macrophages to HDL particles is associated with atherosclerosis in both animals and humans and higher levels of intermediate monocytes are predictive of cardiovascular events in subject with an elevated cardiovascular risk [24,25]. However, in this study, we note that after patients switched to transplantation were excluded from the Cox regression analysis, the relationship between low HDL-C and very early dropout from PD was no longer significant (*p* = .057).

Urine output is considered as an index of residual renal function (RRF). Recent studies have reported that decline of RRF was associated with all-cause mortality in HD patients [26,27] and a higher baseline RRF was an independent factors associated with decreased mortality risk in continuous ambulatory PD patients [28]. Here, we found that a lower volume of 24 h urine output was an important risk factor for very early withdrawal from PD therapy. Moderate decrease in RRF has also been associated with overhydration, peripheral edema and endothelial dysfunction in ESRD patients treated with PD therapy [29,30]. These complications are likely to be associated with an increased risk of switch to HD.

There are several limitations to our current investigation. For one, this is a retrospective cohort study and as such, cannot be used to prove causal relationships. Second, we just used 24 h residual urine volume to evaluate patients RRF. Third, the number of patients who withdrew from PD therapy during the first 90 days was limited and therefore, not all confounding variables were adjusted in our study.

## Conclusions

In conclusion, our data suggest that very early withdrawal from PD therapy is not rare, with death, most commonly due to cardiovascular and infectious diseases, representing the primary reason for very early PD withdrawal. Dialysate leakage and catheter dysfunction were the main reasons for very early transfer to HD, reflecting the need for better evaluation of patients condition before PD catheter insertion and more standardization of the procedure for peritoneal catheter implantation. We found that patients who underwent very early withdrawal from PD were older in age, higher SBP, lower hemoglobin, lower HDL-C and lower residual urine volume at baseline. Medical risk factors management may help prolong PD treatment.

## Supplementary Material

Supplementary Table
