# Supplementary material for: Very early withdrawal from treatment in patients starting peritoneal dialysis
Source: Ren Fail. 2018 Jan 3;40(1):8–14. doi: 10.1080/0886022X.2017.1419965 (PMC6014309; doi:10.1080/0886022X.2017.1419965)
Supplement: Supplementary Table [file IRNF_A_1419965_SM2057.pdf]

Supplementary Table 1. Cox multivariate analysis for death and transfer to HD during the first 90 days of PD treatment

Variable	Univariate Analysis		Multivariate Analysis	
	HR (95% CI)	P Value	HR (95% CI)	P Value
Age (per decade increasing)	1.60(1.32,1.93)	<0.001	1.45(1.18,1.77)	<0.001
Male sex	1.69(0.97,2.93)	0.063	1.29(0.72,2.31)	0.394
Diabetes mellitus (yes/no)	1.58(0.90,2.81)	0.117	0.87(0.47,1.62)	0.868
Cardiovascular disease (yes/no)	1.21(0.67,2.19)	0.526	0.78(0.41,1.47)	0.779
Systolic pressure (per 10mmHg increasing)	1.28(1.13,1.46)	<0.001	1.21(1.06,1.38)	0.006
Hemoglobin (per 10g/l increasing)	0.57(0.49,0.67)	<0.001	0.61(0.50,0.74)	<0.001
ALB (per 1g/L increasing)	0.86(0.81,0.90)	<0.001	0.96(0.89,1.02)	0.210
Serum calcium (per 1mg/dL increasing)	0.08(0.03,0.21)	<0.001	0.53(0.13,2.15)	0.373
Serum phosphorus (per 1mg/dL increasing)	1.07(0.59,1.93)	0.823	0.80(0.46,1.42)	0.452
HDL-C (per 1mmol/L increasing)	0.09(0.03,0.25)	<0.001	0.41(0.17,1.03)	0.057
24h urine output (per 100ml/d increasing)	0.80(0.73,0.87)	<0.001	0.83(0.76,0.91)	<0.001

HE, hemodialysis; PD, peritoneal dialysis; HR, hazard ration; CI, confidence interval; ALB, albumin; HDL-C, high-density lipoprotein cholesterol
